# Histoplasmosis in Non-HIV Infected Patients: Another Neglected Infection in French Guiana

**DOI:** 10.3390/jof10060400

**Published:** 2024-06-01

**Authors:** Houari Aissaoui, Morgane Bourne-Watrin, Benoit Lemarie, Genevieve Guillot, Alolia Aboikoni, Piseth Chhorn, Dana Gaudard, Ghazi Hadj-Amara, Ricardo Manasse, Mahamado Ouedraogo, Charles Salloum, Magalie Demar, Loïc Epelboin, Hatem Kallel, Antoine Adenis, Mathieu Nacher, Kinan Drak Alsibai, Dominique Louvel

**Affiliations:** 1 Department of Medicine, Pneumology and Gastroenterology Units, Cayenne Hospital Center, Cayenne F-97300, French Guiana; houari.aissaoui@ch-cayenne.fr (H.A.); genevieve.guillot@ch-cayenne.fr (G.G.); alolia.aboikoni@ch-cayenne.fr (A.A.); dana.gaudard@ch-cayenne.fr (D.G.); dominique.louvel@ch-cayenne.fr (D.L.); 2 Unité des Maladies Infectieuses Tropicales (UMIT), Cayenne Hospital Center, Cayenne F-97300, French Guiana; morgane.bourne@ch-cayenne.fr (M.B.-W.); loic.epelboin@ch-cayenne.fr (L.E.); 3 Pneumology Department, Cochin University Hospital AP-HP, F-75014 Paris, France; benoit.lemarie@ch-cayenne.fr; 4 Guingamp Hospital Center, F-22200 Pabu, France; piseth.chhorn@ch-cayenne.fr; 5 Department of Pathology, Cayenne Hospital Center, Cayenne F-97300, French Guiana; ghazi.hadjamara@ch-cayenne.fr (G.H.-A.); ricardo.manasse@ch-cayenne.fr (R.M.); 6 Laboratory of Mycology, Cayenne Hospital Center, Cayenne F-97300, French Guiana; mahamado.ouedraogo@ch-cayenne.fr (M.O.); magalie.demar@ch-cayenne.fr (M.D.); 7 Department of Orthopidic Surgery and Neurosurgery, Cayenne Hospital Center, Cayenne F-97300, French Guiana; charles.salloum@ch-cayenne.fr; 8 Intensif Care Unit, Cayenne Hospital Center, Cayenne F-97300, French Guiana; hatem.kallel@ch-cayenne.fr; 9 Amazin PopHealth, Departement of Research and Innovation in Public Health (DRISP), Clinical Investigation Center (CIC Inserm 1424), Cayenne Hospital Center, Cayenne F-97300, French Guiana; antoine.adenis@ch-cayenne.fr (A.A.); mathieu.nacher@ch-cayenne.fr (M.N.); 10 Center of Biological Resources (CRB Amazonie), Cayenne Hospital Center, Cayenne F-97306, French Guiana

**Keywords:** histoplasmosis, non-HIV, neglected infectious disease, French Guiana

## Abstract

(1) Background: Only a few studies on histoplasmosis in immunocompetent patients have been reported in French Guiana. Therefore, we conducted a detailed clinical description of hospitalized patients suffering with histoplasmosis among non-HIV patients. (2) Methods: This is a single-center, retrospective study conducted at Cayenne Hospital Center between 2008 and 2022. (3) Results: Our cohort was composed of 31 (91%) adults (>18 years of age) and 3 (9%) children, with a sex ratio, M:F, of 1:2. The median age was higher among the women than among the men (70 versus 54 years). The collection of respiratory samples constituted the majority of the performed examinations (38%). Fever (>37 °C) was found in 56% of patients. Surprisingly, the histoplasmosis was disseminated in 82% of patients with an overall case fatality rate of 14.7%. However, immunosuppressive conditions were found in 52% (16/31) of the adult patients, including lymphoid hemopathies, diabetes and immunosuppressive drugs. Conclusions: This disease, though rare and usually considered a mostly benign disease in non-HIV patients, presented a relatively high mortality rate in our cohort. Thus, histoplasmosis should be suspected, screened and investigated as a first line of defense in highly endemic areas, even in immunocompetent and non-HIV patients, especially those with fever or chronic respiratory symptoms.

## 1. Introduction

Histoplasmosis, described for the first time by an American pathologist in 1905, is actually one of the most frequent endemic fungal infections in Latin America, notably on the Guiana Shield [1,2,3]. The pathogenic agent is a thermally dimorphic fungus, *Histoplasma capsulatum*. It can persist for years as mold in the soil after its colonization, notably through bird feces and bat guano [4]. Thus, the long survival in the environment offers prolonged opportunities for spores’ inhalation during visits to contaminated areas, causing a primary pulmonary infestation [5].

Pulmonary histoplasmosis is a primary disease with a wide spectrum of clinical manifestations: acute pulmonary histoplasmosis, histoplasmoma and chronic pulmonary histoplasmosis; beyond the lungs, it becomes disseminated and can take acute or chronic forms [1], involving every organ in systemic infections, or it can be the only manifestation when it is focalized in a single organ [1]. Dissemination occurs frequently in immunocompromised patients and is often fatal if not treated [6,7,8,9], but it sometimes also occurs in apparently immunocompetent persons [10].

In French Guiana, an overseas French territory located on the northeast coast of South America, histoplasmosis has been studied since the 1950s. Several studies have described histoplasmosis as the most frequent opportunistic infection in people living with the Human Immunodeficiency Virus (PLHIV) since the use of highly active antiretroviral therapy (HAART) [11,12,13,14]. In French Guiana, it is considered the first AIDS-defining condition, and it used to be the first cause of AIDS-related death [14,15,16,17].

A few clinical cases of histoplasmosis in apparently immunocompetent patients have been reported [18,19,20,21]. Given the high prevalence of HIV in French Guiana, much of the research on histoplasmosis has focused on patients with advanced HIV disease. However, this focus does not imply that the burden of histoplasmosis does not also affect HIV-negative patients. Therefore, given the scarcity of studies on this topic in French Guiana, we conducted a detailed clinical description of hospitalized patients suffering from histoplasmosis among HIV-negative individuals.

## 2. Patients and Methods

This was a single-center, retrospective, observational study conducted at Cayenne Hospital Center from 1 January 2008 to 31 March 2022. Cayenne Hospital is the reference center in French Guiana for the management of tropical infectious diseases. The Parasitology and Mycology laboratory has performed fungal culture since 1997. The Department of Pathology has specialized in tropical infectious disease since 2004, and it systematically performs special stains for cytologic and histologic specimens suspected of fungal infections (May Grunwald Giemsa, Gömöri–Grocott and Periodic Acid Schiff stains).

Files were reviewed based on the Pathology Department records of histoplasmosis, and they were identified by searching for the histoplasmosis code (“B39” within the International Statistical Classification of Diseases and Related Health Problems, “ICD-10”).

The samples collected and studied in the Pathology and Mycology laboratories included fluid samples (respiratory samples with broncho-alveolar lavage (BAL), tracheobronchial aspiration and sputum, ascites, urine, blood, joint fluid and bone marrow aspirate), and tissue biopsies (lung, lymph node, skin, larynx, colon and spinal cord).

The inclusion criteria were as follows: hospitalized patients of any age with an episode of histoplasmosis confirmed according to the European Organization for Research and Treatment of Cancer/Mycosis Study Group (EORTC/MGS) criteria (Histopathology or direct microscopy and/or direct mycological examination or mycological culture of specimens obtained from an affected site showing the distinctive form of the fungus) [22]. First episodes and relapses of histoplasmosis infection were all included.

Isolated positive PCR or serology and unproven histoplasmosis (improvement after empirical antifungal therapy) were excluded. A positive serology for HIV was an exclusion criterion.

Socio-demographic, clinical, biological (standard biological examinations, mycology and pathology on any type of samples), radiologic and therapeutic data were collected from medical records on a standardized file.

### 2.1. Statistical Analysis

The collected data were then processed using Microsoft Excel^®^ 2019—version number 1808, for statistical analysis. Frequencies and proportions were calculated for categorical variables. For quantitative variables, means, medians and interquartile ranges [Q1; Q3] were reported.

### 2.2. Ethical Statement

The project consisted of reutilizing available personal health data; thus, it was classified as research not involving human persons and conducted according to reference methodology n°004 of the Commission Nationale Informatique et Libertés (CNIL) in compliance with the General Data Protection Regulation (EU 2016/679). A letter of information was sent to all participants to inform them of the collection of their data and the purpose of the study.

## 3. Results

### 3.1. Epidemiological Data

A total of 34 hospitalized patients from the Medicine, Intensive Care Unit (ICU), Infectious Tropical Diseases Unit (ITDU), Dermatology and Paediatrics Departments were included in our cohort. The cohort was composed of 91% (31/34) adults (≥18 years of age) and 9% (3/34) children (Table 1), with a sex ratio, M:F, of 2. The median age was higher among the women than among the men (70 versus 54 years). The patients were originally from French Guiana (47%), Mainland France (21%) and Brazil (18%). Patients from Surinam and Haiti were less represented (6 and 3% respectively). Nearly half of the patients (48%) lived in Cayenne and its surroundings, including Cayenne, Rémire-Montjoly and Matoury, the most inhabited areas of French Guiana.

### 3.2. Clinical Presentation

All cases were first episodes *Histoplasma capsulatum* infection. General condition was impaired in 62% of patients with a WHO performance score >2. Fever (>37 °C) was found in 56% of patients.

In 82% of the cases, the histoplasmosis was disseminated: acute disseminated histoplasmosis accounted for 8 cases (23%), and chronic disseminated histoplasmosis accounted for 20 cases (59%), in contrast with 18% of cases in with pulmonary localization: acute pulmonary histoplasmosis in one case (3%) and chronic pulmonary histoplasmosis in five cases (15%).

**Figure 1:**
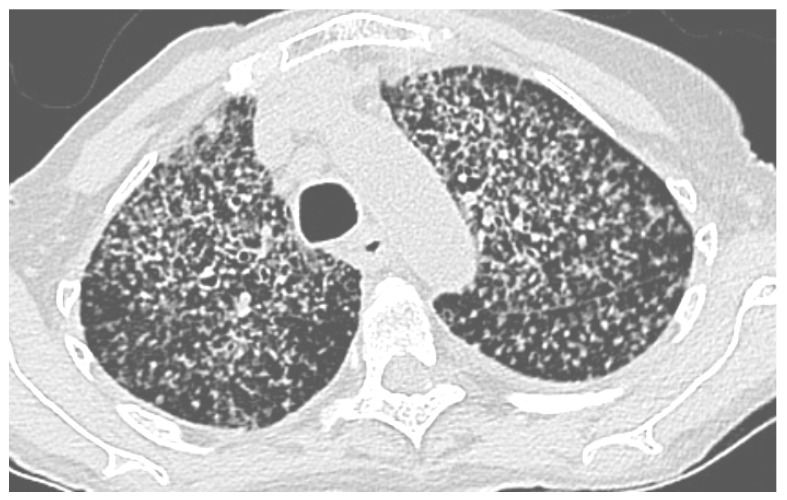
Chest CT scan, miliary histoplasmosis in 91-year-old woman occurring after cleaning droppings, particularly those of bats, in a confined place without protection. She unfortunately died despite treatment. Scale bar: 25%.

The pulmonary features found in thoracic computerized tomography (CT) scans varied. They presented as diffuse miliary micronodules (four cases) (Figure 1) or non-diffuse micronodules (one case). Bilateral pulmonary nodules with regular contours (one case), localized ground glass nodules (one case), a calcified nodule (one case), alveolar pulmonary condensations (four cases) and excavated condensations (two cases) were also found via CT scan. In one case, the pulmonary histoplasmosis coexisted with a non-small-cell lung carcinoma in the same lung lobe.

Regarding digestive involvement, focal colonic involvement (without evidence of other localization) was the most frequent. The four cases of colonic histoplasmosis presented as ulcerations upon endoscopy (unique ulceration localized in the coecum in one case and multiple ulcerations spread throughout the colon in three cases).

**Figure 2:**
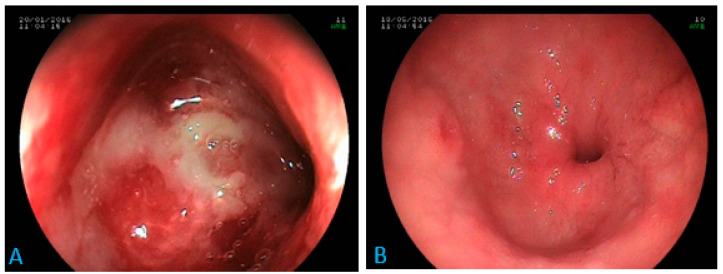
Digestive endoscopy: inflammatory stenosis at the tip of the duodenal bulb in a 70-year-old patient (**A**) suffering from epigastric pain, anorexia and weight loss. Diagnosis of histoplasmosis via biopsy with favorable evolution under antifungal treatment (**B**). Scale bar: 100%.

Ascites were found in both focal and disseminated forms. Gastric or hepatic involvements were found only in disseminated forms. One case of isolated duodenal involvement, as a tight stenosis of the duodenal bulb (Figure 2), was found.

Only one case of cerebral involvement was found in our cohort in a severely malnourished 10-year-old child suffering from disseminated histoplasmosis. Similarly, one oral mucosa histoplasmosis was found in a 79-year-old man in the context of disseminated histoplasmosis after anticancer chemotherapy.

Immunosuppressive conditions were found in 52% (16/31) of adult patients: immunosuppressive or biologic agents (long-term corticosteroid therapy, anticancer chemotherapy, methotrexate and anti-TNF alpha), malignant lymphoid hemopathies and solid organ cancer, kidney transplant, autoimmune disease, diabetes, hepatitis cirrhosis, alcoholism and HTLV-1 infection (Table 2). In our cohort, four patients had between two and three immunosuppressive pathologies. The three pediatric cases were all chronic disseminated forms. In immunosuppressed, non-HIV histoplasmosis patients, all forms were disseminated.

We found five cases of histoplasmosis in patients with lymphoid malignancies. In four of these cases, *Histoplasma capsulatum* fungemia was diagnosed via blood cultures in the presence of fever during the course of chemotherapy.

**Figure 3:**
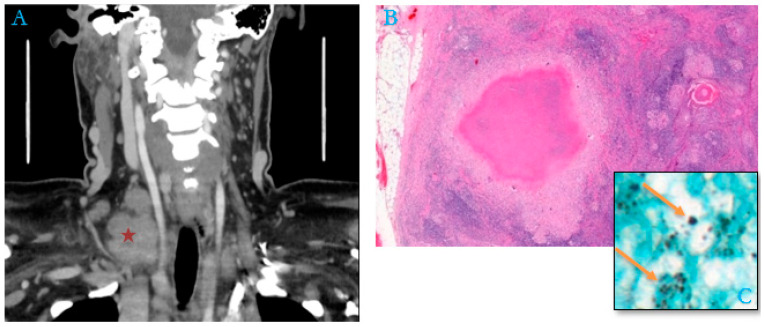
Package of right supraclavicular adenopathy (red asterisk) in a 51-year-old woman, a housemaid with no previous history (**A**). (**B**) Histological section of lymph node shows numerous granulomas with epithelioid cells associated with multinucleated giant cells, centered by caseous necrosis mimicking tuberculoid granuloma (Hematoxylin–Eosin–Safran, “HES,” Stain, ×200). (**C**) Numerous extracellular and intra-histiocytic yeasts of *Histoplasma capsulatum* (Gömöri–Grocott stain, orange arrows, ×100).

By contrast, in 48% of the adult cases (15/31), no cause of immunosuppression or associated conditions was mentioned in the patient’s medical record (an example in Figure 3).

### 3.3. Biological Data

An inflammatory syndrome with elevated CRP was found in 91% of cases with a median of 45 mg/L. Anemia (hemoglobin level < 11.5 g/dL), thrombocytopenia (platelets < 150 G/L) and neutropenia (neutrophils < 2 G/L) were found in 51.5%, 31% and 9% of cases, respectively. Aspartate amino transaminase (AST) was increased (>35 Ui/L) in 42% of cases and alanine amino transaminase (ALT) in 19.5% of cases. The greatest disturbances were found in GGT in 55% of cases (up to 19 times the upper normal level). LDH levels were elevated in 37% of cases (up to eight times the upper normal level).

### 3.4. Diagnostic Methods

Respiratory samples accounted for the majority of the examined specimens (38%), including 69% of BAL. In addition to being the main examination performed in the respiratory tract, BAL was, in fact, the most frequently performed examination in general (26.5% of all specimens). Fungal blood cultures came in second place (20.5%), followed by digestive biopsies (15%). The puncture of ascites fluid and lymph node biopsies represented 9% each.

Concerning the analyses requested by the physicians, mycological examination was the most frequently requested (88% of cases) with 82% of positive *Histoplasma capsulatum* cultures. Cytological analysis was less frequently requested (20.5%), but it was nevertheless frequently helpful. For example, a cytological examination was requested for 85% of the performed BALs, allowing the rapid detection of *Histoplasma capsulatum* in 54% of cases (Table 3).

### 3.5. Treatment Data

Antifungal therapy was initiated in 24/34 patients (71%), with Itraconazole monotherapy upfront (17/24) and Ambisome relayed with Itraconazole (7/24). Treatment was completed for only 18/24 patients. The duration of treatment varied from one patient to another: 2 months for two patients, 3 months for five patients, 4 months for one patient, 6 months for seven patients, 12 months for two patients and 20 months for one patient. Among them, the prescribed dose of Itraconazole varied from 400 mg for nine patients (50%) to 200 mg for six patients (33.3%), 100 mg for two patients (11.2%) and 300 mg for one patient (5.5%).

Five patients out of 34 died from histoplasmosis, including four patients who died in the ICU, leading to an overall case fatality rate of 14.7%. All five patients who died of histoplasmosis in our cohort, aged 36, 45, 60, 65 and 93, were diagnosed early and treated for disseminated histoplasmosis.

Four patients who died in the ICU presented an acute form of histoplasmosis with multi-visceral failure. One of these cases (a patient aged 36 years) had septic shock with progressive, disseminated histoplasmosis.

Two of these four patients had immunosuppressive pathologies (chronic alcoholism with HTLV1 infection and kidney transplantation with immunosuppressive drugs).

## 4. Discussion

This 14-year study identified 34 cases of histoplasmosis in non-HIV patients requiring hospitalization and treatment. This small sample is similar to that presented in studies carried out in neighboring countries in the same geographical area of the Amazon with an endemic presence of histoplasmosis, such as Brazil [2] and Peru [17]. On the other hand, although the exact incidence of mild or non-symptomatic histoplasmosis in endemic areas in French Guiana or in other neighboring countries of South America is unknown, histoplasmin skin test prevalence used in the early of 1950s allowed for the revelation of the extent of *Histoplasma capsulatum* infection in many areas [23]. A previous study supposed that 90% of the tested population in some parts of the USA (endemic arears of Mississippi and the Ohio River valleys of Missouri) had been exposed to *Histoplasma* by the age of ≥15 years [24]; hundreds of thousands of individuals in these endemic areas are infected each year and remain asymptomatic [1]. In French Guiana, 32.5% of the general population in the early 1950s was infected or had contact with *Histoplasma capsulatum* [25]. However, these skin tests are no longer marketed today because of cross-reactions with other fungi [1].

In persons with HIV, histoplasmosis takes a progressive, disseminated form in more than 95% of patients [26]. Although much research has been performed in the context of HIV, the problem of histoplasmosis extends beyond the realm of HIV, albeit with a much lower incidence.

*Histoplasma capsulatum* infection is usually due to inhalation of spores contained in enriched soils, which constitute the only reservoir of this mold [1]. Therefore, any domestic or professional activity in endemic regions can lead to the aerosolization of these spores, favoring inhalation and infection [1]. The occurrence of infection after the inhalation of spores depends on the inhaled inoculum and the immune response of the host [22].

The endogenous reactivation of previously inhaled spores that have remained quiescent yeasts in the host organism, similar to what is known for tuberculosis, has been suggested [7,8,9,11,12,13,14,15,16,17,18,19,20,21,22,24,25,26,27] and could explain certain forms of histoplasmosis occurring in immunocompetent patients several months after their return from a trip to an endemic area. However, in French Guiana, data from HIV cohorts have shown a substantial seasonal component, suggesting de novo infections [28].

In comparison with previous data in HIV patients infected with histoplasmosis [16], the sex ratio, M:F, appears identical to our study at 2, but the median age of onset in males and females was greater in the non-HIV population than in PLHIV (54–70 for non-HIV versus 41.4–37.5 for HIV patients). Patients from French Guiana were the most represented in both groups, but patients from Haiti were the second most represented among PLHIV, probably due to the high prevalence of HIV infection in Haiti. In our HIV-negative cohort, patients from mainland France represented the second most frequent group among non-HIV patients, followed by patients from Brazil.

In nearly 48% of cases, the patients were apparently immunocompetent. However, it is important to mention that no specific investigation of underlying immunosuppression was performed in these patients (serum protein electrophoresis, determination of immunoglobulin class, lymphocyte immunophenotyping, etc.), as the patients did not show patent signs of immunosuppression, which could be considered a limitation in this study.

Thus, in French Guiana, HIV infection remains the main immunosuppressive condition that favors histoplasmosis, especially since it is one of the most prevalent forms of immunosuppression, and it is mostly discovered at a late stage in nearly one-third of cases [29], with heterogeneous clinical presentations [30] most often fatal in the absence of appropriate treatment [31].

In our study, the immunosuppression-associated conditions—in particular lymphoid malignancies or treatment with long-term corticosteroids, chemotherapies or immunosuppressant drugs—are causes of cellular immunity impairment similar to the HIV-mediated immune impairment, highlighting the role of cell-mediated immunity against the dissemination of histoplasmosis beyond the pulmonary barrier. [1].

The analysis of cases of *Histoplasma capsulatum* fungemia should pay particular attention to the occurrence of acute febrile peaks during chemotherapy treatment, especially in hematologic lymphoid malignancies, and should lead to a search for histoplasmosis in the initial investigation.

Adrenal involvement is the most frequently described in the literature in immunocompetent patients [12], especially in Asia [32,33,34], as a bilateral adrenal hypertrophy or adrenal calcifications on CT scan [32]. This localization can sometimes lead to adrenal insufficiency suggestive of Addison’s disease or tuberculosis [34]. In our cohort of non-HIV patients, no morphological abnormalities of the adrenals in imaging were reported, and no clinico-biological signs of adrenal insufficiency were found. In HIV-infected patients in French Guiana, a low rate of 0.85% of adrenal damage via histoplasmosis was previously described [12]. Perhaps there again, the immunosuppression circumstances explain why it is rare, by contrast with localized adrenal presentations, where the host is immunocompetent but the local immunosuppression due to the adrenal hormonal environment creates a niche for infection.

Biology findings in non-HIV patients with histoplasmosis were non-specific (Table 4). In PLHIV with histoplasmosis, a platelet count of less than 100,000/mm^3^ and an LDH level greater than twice the normal level were defined as severity factors [13]. In our study, we calculated the mean LDH level for the five severe cases of patients who died from histoplasmosis, and we found an increase in this level to four times the normal value threshold. The median platelet count was normal at 227 G/L. However, cytolytic impairment predominantly via AST and cholestasis predominantly via GGT were found in a comparable manner to HIV [35].

Regarding the analyzed specimens (Table 3), respiratory samples constituted the majority of the performed examinations (38% of samples). BAL accounted for 69% of these respiratory specimens with a good sensitivity compared to other examinations (71% positive direct examination in mycology, 71% in culture and 54% in cytology).

Cultures dominated all other diagnostic methods, confirming their place as the gold standard in the diagnosis of histoplasmosis. Few cytological examinations were performed, despite their potential contribution, in line with the same results found in disseminated histoplasmosis in PLHIV [5].

Concerning the therapeutic data, we found a lot of missing information concerning the follow-ups and outcomes of patients’ treatments, in contrast to the cohort of PLHIV. In French Guiana, specialized care structures dedicated to PLHIV have allowed a better organization of their treatment and follow-up, particularly the establishment of an HIV cohort and an HIV histoplasmosis database created in 1992 [5,8,12].

Itraconazole was the main antifungal agent used in the treatment of histoplasmosis, but its dosage and duration of treatment varied considerably from one patient to another, without any conformity with the guidelines [9,14]. Thus, it appears that the management of histoplasmosis in PLHIV is better codified and more consistent. All these data showed that, while histoplasmosis is considered a neglected infection in Latin America [15], it seems even more neglected in non-HIV and immunocompromised patients. While there is a growing recognition that febrile HIV-infected persons should be suspected of potentially having histoplasmosis, there is no such heuristic for patients without HIV, and clinicians should be vigilant.

## 5. Conclusions

In endemic areas such as French Guiana, symptomatic histoplasmosis in non-HIV patients appears to be a relatively rare disease, affecting both apparently immunocompetent individuals and those with comorbidities and immunosuppressive pathologies. In our study, the majority of hospitalized non-HIV patients with comorbidities had mainly lymphoid malignancies or diabetes, or had been treated with immunosuppressive drugs. This disease, though rare and usually considered a mostly benign disease in non-HIV patients, presented a relatively high mortality estimated at 14.7% in our cohort. Thus, histoplasmosis should be suspected, screened and investigated as a first line of defense in highly endemic areas, even in immunocompetent patients, especially those with fever or chronic respiratory symptoms.

## Figures and Tables

**Table 1:** Epidemiological and clinical characteristics of non-HIV patients diagnosed with histoplasmosis.

**Place of Birth:**	**Number/% of patient**
French Guiana	16 (47)
Mainland France	7 (21)
Brazil	6 (18)
Suriname	2 (6)
Venezuela	1 (3)
Haiti	1 (3)
Italy	1 (3)
**Sex:**	**Number/% of patient**
Men	23 (68)
Women	11 (32)
Sex ratio:	2
**Age:**	**Number/Median**
Children	3 (7)
Men	21 (54)
Women	10 (70)
**General Condition:**	**Number/% of patient**
WHO performance status score > 2	21 (62)
WHO performance status score ≤ 2	11 (32)
Not defined	2 (6)
	**Number/% of patient**
**Fever**	19 (56)
**Clinical Forms:**	**Number/% of patient**
Acute disseminated histoplasmosis	8 (23)
Chronic disseminated histoplasmosis	20 (59)
Acute pulmonary histoplasmosis	1 (3)
Chronic pulmonary histoplasmosis	5 (15)

**Table 2:** This table shows the number of immunosuppressive pathologies in non-HIV patients when histoplasmosis had been diagnosed.

Immunosuppressive Conditions and Diseases Associated with Histoplasmosis	Number of Patients
Malignant lymphoid hemopathies	5
Diabetes	5
Immunosuppressive drugs	7
Biologic agents (anti-TNF alpha)	1
Advanced hepatitis/cirrhosis	2
Solid organ cancer	2
Autoimmune disease	3
HTLV-1 infection	1
Alcoholism	3

**Table 3:** Diagnostic methods of histoplasmosis in non-HIV patients.

Specimen	Patients Tested *n* (%)	Positive Direct Examination (%)	Positive Culture (%)	Positive Cytology (%)	Positive Histology (%)
Respiratory samples:	13/34 (38)			6/11 (54)	
- BAL	9/13 (69)	5/7 (71)	5/7 (71)		
- Tracheobronchial suctioning	1/13 (7.6)	1/1 (100)	1/1 (100)		
- Sputum	2/13 (15)	1/2 (50)	1/2 (50)		
- Biopsy	1/13 (7.6)	--	--		1/1 (100)
Blood culture	7/34 (20.5)	--	7/7 (100)	--	
Digestive tract biopsy	5/34 (15)	3/4 (75)	3/4 (75)	--	4/4 (100)
Ascites	3/34 (9)	2/3 (66)	3/3 (100)	--	
Lymph node biopsy	3/34 (9)	0/3 (0)	1/3 (33)	--	2/3 (66)
Bone marrow aspirate and biopsy	2/34 (6)	--	1/2 (50)	--	1/2 (50)
Urine	2/34 (6)		2/2 (100)	--	--
Laryngeal biopsy	2/34 (6)	1/2 (50)	2/2 (100)	--	1/2 (50)
Cutaneo-mucous biopsy	2/34 (6)	2/2 (100)	2/2 (100)	--	
Synovial fluid	1/34 (3)	--	--	1 (100)	--

**Table 4:** Biological data found in non-HIV patients with histoplasmosis.

Laboratory Tests	N_0_/Total/(%)	Median	[Q_1_; Q_3_]
Hemoglobin level	--	11.4	[8.8; 12.7]
<11.5 g/dL	17/33 (51.5)		
Neutrophil count	--	4.6	[3.0; 6.1]
>7 G/L	6/32 (18)		
<2 G/L	3/32 (9)		
Platelets	--	227	[130.0; 332.0]
<150 G/L	10/32 (31)		
Albumin	--	30.5	[25.5; 33.5]
<35 g/L	18/23 (78)		
Protein	--	65	[58.0; 78.0]
<60 g/L	10/29 (34.5)		
Creatinine level	--	84.25	[62.5; 127.2]
>100 umol/L	11/32 (34.5)		
Aspartate aminotransferase (ASAT) level	--	30	[20.5; 70.5]
>35 U/L	13/31 (42)		
Alanine transaminase (ALAT) level	--	30	[12.5; 44.0]
>55 U/L	6/31 (19.5)		
Alkaline phosphatase level	--	107.5	[70.0; 181.7]
>150 U/L	8/31 (26)		
Gamma glutamyltransferase (GGT) level	--	54	[25.5; 136.5]
>50 U/L	17/31 (55)		
Lactate dehydrogenase (LDH) level	--	262	[201.0; 394.0]
>300 U/L	10/27 (37)		
C-reactive protein level	--	45.1	[18.5; 100.5]
>5 mg/L	29/32 (91)		

## Data Availability

Data are contained within the article.
